# Chronic Kidney Disease, Fluid Overload and Diuretics: A Complicated Triangle

**DOI:** 10.1371/journal.pone.0159335

**Published:** 2016-07-21

**Authors:** Yusra Habib Khan, Azmi Sarriff, Azreen Syazril Adnan, Amer Hayat Khan, Tauqeer Hussain Mallhi

**Affiliations:** 1 Discipline of Clinical Pharmacy, School of Pharmaceutical Sciences, University Sains Malaysia, Penang, 11800, Malaysia; 2 Chronic Kidney Disease Resource Centre, School of Medical Sciences, Health Campus, University Sains Malaysia, Kubang Kerain, 16150, Kelantan, Malaysia; University Medical Center Utrecht, NETHERLANDS

## Abstract

**Background:**

Despite promising role of diuretics to manage fluid overload among chronic kidney disease (CKD) patients, their use is associated with adverse renal outcomes. Current study aimed to determine the extent of renal deterioration with diuretic therapy.

**Methods:**

A total 312 non-dialysis dependent CKD (NDD-CKD) patients were prospectively followed-up for one year. Fluid overload was assessed via bioimpedance spectroscopy. Estimated GFR (eGFR) was calculated from serum creatinine values by using Chronic Kidney Disease- Epidemiology Collaboration (CKD-EPI) equation.

**Results:**

Out of 312 patients, 64 (20.5%) were hypovolemic while euvolemia and hypervolemia were observed in 113 (36.1%) and 135 (43.4%) patients. Overall 144 patients were using diuretics among which 98 (72.6%) were hypervolemic, 35 (30.9%) euvolemic and 11 (17.2%) were hypovolemic. The mean decline in estimated GFR of entire cohort was -2.5 ± 1.4 ml/min/1.73m^2^ at the end of follow up. The use of diuretics was significantly associated with decline in eGFR. A total of 36 (11.5%) patients initiated renal replacement therapy (RRT) and need of RRT was more profound among diuretic users.

**Conclusions:**

The use of diuretics was associated with adverse renal outcomes indicated by decline in eGFR and increasing risk of RRT initiation in our cohort of NDD-CKD patients. Therefore, it is cautiously suggested to carefully prescribe diuretics by keeping in view benefit versus harm for each patient.

## Introduction

Chronic kidney disease (CKD) is a global health concern that substantially increases the risk of mortality and the use of specialized health care [1]. Progressive loss of renal function causes reduced sodium filtration and inappropriate suppression of tubular reabsorption that ultimately lead to volume expansion [2]. Fluid overload frequently manifests in patients with moderate to particularly late stages of CKD and has been associated with hypertension, congestive heart failure (CHF), left ventricular hypertrophy (LVH) as well as edema. In such cases, diuretics are frequently prescribed to control blood pressure and for symptomatic relief of fluid overload [3, 4]. However, the role of diuretics remains quite controversial in CKD patients. Apart from their beneficial effects, these agents also decrease glomerular filtration rate (GFR) and cause metabolic disturbances that in turn increases risk of cardiovascular events [5, 6].

Various guidelines suggest the use of loop (GFR<30ml/min/1.73m^2^) and thiazide diuretics (GFR >30ml/min/1.73m^2^) in CKD patients [7]. Unfortunately, randomized controlled trials demonstrating clinical benefits and subsequent harms of diuretic therapy in mild to moderate CKD patients do not exist. Observational studies with small sample size and short duration have shown that diuretics decrease blood pressure (BP) and improve edema in CKD patients but their use, particularly at higher doses, is associated with rise in serum creatinine and several metabolic complications [4,8–9].

The clinical assessment of fluid overload is relatively difficult and diuretics are mostly prescribed in clinical settings on the basis of high blood pressure and physical signs of edema. Although edema can roughly estimate excess extravascular volume but it is of limited value in assessing excess intravascular volume. Moreover, several liters of water should be retained before physical signs of edema become visible [10]. Other techniques to assess fluid status include ultrasonic evaluation of inferior vena cava diameter but it is subjected to interpatient and interoperator variability. Biomarkers such as brain natriuretic peptide (BNP) and N-terminal pro brain natriuretic peptide (NT-pro BNP) can reflect changes in fluid status but both are influenced by presence of cardiovascular disease (CVD) and are also accumulated in CKD patients, rendering these methods inappropriate for evaluation of fluid status in CKD patients [11].

Recently few studies have used bioimpedance spectroscopy i.e. Body Composition Monitoring (BCM) for assessment of fluid status in CKD patients and have shown association of fluid overload with decline in renal function in non-dialysis dependent (NDD) CKD patients [2, 11–13]. However, all except one have not addressed the use of diuretics and its association with both volume overload and decline in renal function [4]. In order to overcome this clinical issue, we conducted a prospective observational study to assess association of diuretics use with severity of fluid overload and loss of renal function/decline in eGFR. The purpose of current study was not to devalue the potential benefits of diuretic therapy among CKD patients. We intended to see the extent of eGFR decline and odds of RRT initiation among NDD-CKD patients receiving diuretics. The findings of the present study could be hypothesis-generating, forming evidence to be considered during future research.

## Methodology

### Study location and Participants

**Fig 1:**
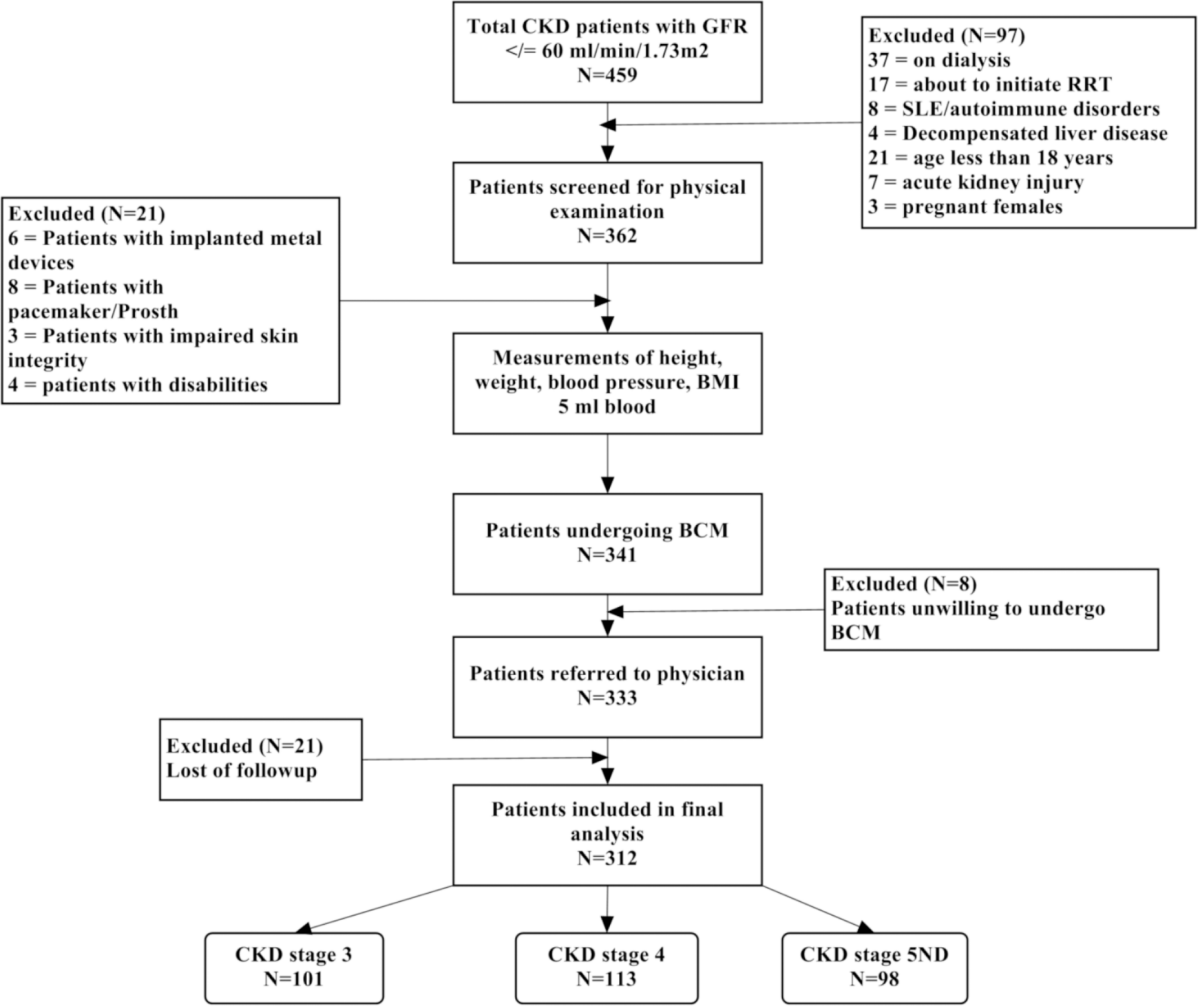
Methodological flow chart of study.

Current study was conducted at a tertiary care hospital in North-Eastern part of Malaysia. All patients visiting outpatient nephrology clinic with confirm diagnosis of CKD (stage 3 to 5ND i.e. non dialysis) according to K/DOQI were invited to participate in the study. Subjects with active infection, decompensated liver disease (liver cirrhosis, ascites), acute kidney injury and autoimmune disorders i.e. systemic lupus erythematous (SLE) were excluded on account of rapid decline in eGFR (>5ml/min/1.73m^2^). Moreover, participants with pregnancy, malignancies, prosthesis, pacemakers, implanted metal devices (vales, stents or sutures), disabilities and impaired skin integrity were excluded from study due to pre- requirements of BCM (Fig 1). Subjects with uncontrolled hypertension (BP >180/110 mmHg), urinary tract obstruction, unstable CKD (having >15% increase in serum creatinine over last 3 months), congestive heart failure, allergy to sulfa drugs and using diuretics or NSAIDs at the time of beginning of study were excluded at the preliminary stage (before physical examination) of patient recruitment.

### Ethical consideration

Current study was approved by ethical committee [Jawatankuasa Etika Penyelidikan-Manusia (JEPeM] of Hospital University Sains Malaysia. All the patients were asked to sign a consent form before participating in study. Identity of each patient was kept confidential and patients were anonymized during data analysis.

### Data collection

Patient demographics were recorded from hospital database and by in person interviewing. A physical examination was performed to measure height, weight and blood pressure. Blood pressure was recorded as mean of three consecutive measurements with 5 minutes interval, using one single calibrated manual sphygmomanometer. Comorbidities were defined as follow: diabetes; repeated determination of fasting plasma glucose (>6.4mmol/L), high blood pressure; an average systolic/diastolic blood pressure of ≥140/90mm Hg or prescription of anti-hypertensive medications, hyperlipidemia; low density lipoprotein (LDL)level of >100 mg/dl and cholesterol level >200mg/dl, Cardiovascular disease (CV); CV disease includes coronary artery disease or ischemic heart disease (blockage of coronary artery by artherosclerosis that results in reduced blood supply to heart) and includes stroke, angina and myocardial infarction. Body mass index (BMI) was subsequently calculated as weight/height (kg/m^2^). Following an overnight fasting, 5 ml of blood was withdrawn to measure full blood count, serum creatinine, calcium, phosphorous, albumin and lipid profile. Estimated GFR (eGFR) was calculated from serum creatinine values by using Chronic Kidney Disease- Epidemiology Collaboration (CKD-EPI) renal function predictive equation [14]. Severity of leg edema was graded on a 4-point scale as: 0 (none), 1 (mild), 2 (moderate), 3 (severe) via assessment chart used in hospital. After routine examination, patient then underwent measurement of fluid status by means of Bioimpedance spectroscopy followed by their scheduled check-up at clinic. Both patients and consulting physicians were unaware of BCM results. Prescription of diuretics was then checked via Hospital online prescription database by entering patient code. All patients were followed up at outpatient nephrology clinic at 3 months interval to ascertain vital status and renal function for a period of one year.

### Measurement of Fluid status

A multi frequency (5–1000kHz) bioimpedance spectroscopy device (Body composition Monitor, BCM, Fresenius Medical Care, Germany) was used to assess fluid status. The principle of measuring current flow through body is dependent on applied frequency. At low frequencies, current predominantly passes through extracellular space while at higher frequencies it passes through both intracellular and extracellular water. This device has been intensively validated against different gold standards in general and hemodialysis population [10,15,16]. However few studies have shown its validation in NDD-CKD population [11–13,17]. The measurements were performed after a 5 minute resting period with patient lying in the supine position. Electrodes were attached to one foot and one hand at the ipsilateral side. Metal and electronic devices that might interact with transmission were removed before start of procedure. Furthermore, the procedure was performed after ensuring that patient had not consumed a heavy meal at least within 4 to 5 hour, had not exercised within 12 hours prior to test and had not consumed any beverages including alcohol and caffeine within 24 hours before the test. In the present study, absolute overhydration/fluid overload (OH), intracellular water (ICW), extracellular water (ECW) and total body water (TBW) were calculated based on bioelectrical impedance analysis following the model of Moissl et al [18]. The value of overhydration (OH) as calculated by BCM was used as an indicator of fluid overload. In general population, the 90^th^ percentile of OH is +1.1 L. Accordingly, OH > 1.1L was classified as fluid overload (hypervolemia). Hypovolemia was defined as OH value lower than 10^th^ percentile -1.1 L. An OH value between ± 1 was defined as euvolemia i.e. normal hydration status [19, 20]. Bioimpedance analysis (BIA) was done twice i.e. at the time of beginning of study and at the end of follow-up (1 year).

### Statistical analysis

The accordance of quantitative data with normal distribution was examined with the Kolmogorov-Smirnov test. Continuous data were presented as mean (standard deviation). Categorical data were presented as frequency (proportion) for which frequency served as numerator and total number of patients served as denominator. Relevant denominator was stated before proportion, where it varied. Comparison of categorical variables between two groups was done by using Chi-Square test (if at least 80 percent of cells have expected frequencies of 5 or more) or Fisher`s Exact test (if less than 80 percent of cells have expected frequencies of 5 or more). Comparison of continuous variables was done by a Student’s t-test when comparing two groups and by ANOVA when comparing more than two groups. Decline in kidney function was assessed by eGFR slope, defined as regression coefficient between eGFR and time.

Univariate correlation between eGFR decline and potential explanatory variables was assessed by Pearson Correlation analysis. Correlations were analyzed to determine strength of relationship between continuous variables. Significant variables in univariate were then entered into multivariate regression analysis to identify variables that are independently associated with decline in eGFR. By keeping overhydration (OH), blood pressure and baseline eGFR as continuous variable, univariate and multivariate cox regression analysis was done to find hazard ratio (HR) of diuretic users and non-users in terms of adverse renal outcomes (initiation of RRT, decline in eGFR). For all analysis a p-value of <0.05 was considered statistically significant. All categorical data were entered by coding 0 indicating absence/ no while 1 showing presence/yes. Data were analyzed by using SPSS 20.0.0.

## Results

Baseline demographics and clinical characteristics of study participants for each category of fluid status are shown in Table 1. After applying exclusion criteria, a total of 312 clinically stable NDD-CKD patients (mean age 64.5 ± 6.43, 57% male) were enrolled in current study. All patients had moderate to severe CKD with mean eGFR 21.4 ± 9.2 ml/min/1.73 m^2^ (32%, 36% and 31% in CKD stage 3, 4, 5ND respectively). About 81% of study participants were hypertensive and 64% were diabetics. Pre-existing and documented cardiovascular and cerebrovascular disease were noted in 29% and 10% patients, respectively.

**Table 1:** Clinical characteristics of CKD patients stratified by OH values.

Demographics	Overall N = 312	Hypovolemic N = 64	Euvolemic N = 113	HypervolemicN = 135	p-value
Age (years)	64.5 ± 6.43	67.0 ± 7.68	62.3 ± 5.81	65.1 ± 6.73	0.2
Male gender	178 (57.0%)	28 (43.8%)	57 (50.4)%	93 (68.9%)	0.001
Body mass index (kg/m^2^)	24.1 ± 5.4	24.3 ± 3.8	23.7 ± 3.8	24.6 ± 3.9	0.5
Current Smoker	96 (30.7%)	20 (31.2%)	27 (23.8%)	49 (36.2%)	0.1
Current alcohol drinkers	0	0	0	0	
**Comorbidities**					
Hypertension	252(80.7%)	44(68.7%)	89(78.7%)	119(88.1%)	0.01
Diabetes mellitus	200 (64.1%)	28 (43.7%)	66 (58.4%)	106 (78.5%)	<0.001
Hyperlipidemia	166 (53.2%)	35 (54.6%)	55 (48.6%)	76 (56.2%)	0.8
Cardiovascular disease	90 (28.8%)	14 (21.8%)	30 (26.5%)	46 (34.0)%	0.04
Cerebrovascular disease	33 (10.6%)	3.0 (4.6%)	12 (10.6%)	20 (14.8%)	0.003
**CKD staging**					
Stage 3	101 (32.4%)	34 (53.1%)	50 (44.2%)	17 (12.6%)	<0.001
Stage 4	113 (36.2%)	22 (34.4%)	42 (37.2%)	49 (36.3%)	0.03
Stage 5ND (non-dialysis)	98 (31.4%)	8.0 (12.5%)	21 (18.6%)	69 (51.1%)	<0.001
eGFR ml/min/1.73m^2^	21.4 ± 9.2	27.1 ± 9.9	27.0 ± 10.3	16.4 ± 9.6	0.03
Systolic BP(mmHg)	140.4 ± 21.3	133.5 ± 15.4	138.2 ± 18.7	147.8 ± 10.1	<0.001
Diastolic BP(mmHg)	74.5 ± 10.1	75.4 ± 9.1	73.3 ± 10.3	75.2 ± 11.4	0.6
Leg edema score>1	72 (23.0%)	3 (4.6%)	13 (11.5%)	56 (41.4%)	<0.001
**Body composition parameters**					
Lean tissue index(kg/m2)	13.9 ± 3.1	14.2 ± 2.8	13.5 ± 2.5	13.8 ± 2.7	0.04
Fat tissue index (kg/m2)	9.6 ± 3.5	10.3 ± 4.1	9.7 ± 3.8	9.4 ± 3.5	0.07
Total body water (TBW) L	34.9 ± 5.4	33.0 ± 5.8	34.3 ± 5.6	37.4 ± 7.6	0.001
Intracellular water (ICW) L	18.2 ± 3.4	17.6 ± 3.4	18.4 ± 3.3	18.5 ± 4.2	0.7
Extracellular water(ECW) L	16.7 ± 3.7	15.4 ± 2.4	15.9 ± 2.9	18.9 ± 4.1	<0.001
ECW: ICW ratio	0.91 ± 0.2	0.87 ± 0.2	0.86 ± 0.2	1.02 ± 0.2	0.001
Fluid overload (OH) L	1.0 (0.5–2.3)	-1.6 (-1.2 - -2.3)	1.0(-0.4–0.9)	2.9 (2.2–4.2)	<0.001
**Laboratory profile**					
Na (mmol/L)	138.54 ± 3.35	138 ± 3.4	138.40 ± 3.2	140.67 ± 3.0	0.4
K (mmol/L)	4.36 ± 0.53	4.36±0.59	4.32 ± 0.48	4.96 ± 0.45	0.3
Urea (mmol/L)	10.21 ± 5.77	8.81 ± 3.69	8.84 ± 4.78	12.07 ± 6.31	0.04
Uric acid (mg/dl)	7.9 ± 1.5	7.6 ± 1.4	7.9 ± 1.9	8.2 ± 1.8	0.02
Ca-phosphate product (mg^2^/dl^2^)	37.3	37.7	36.5	37.9	0.6
Albumin (g/dl)	4.2 ± 0.4	4.4 ± 0.5	4.3 ± 0.6	4.0 ± 0.3	0.03
Fasting blood sugar (g/dl)	101	101	99	106	0.4
Glycated hemoglobin (%)	5.7	5.6	5.6	6.1	0.03
Cholesterol (mg/dl)	182 (155–212)	188 (164–216)	183 (160–212)	175 (141–204)	0.03
Triglycerides (mg/dl)	119 (83–169)	126 (90–167)	109 (80–172)	115 (75–161)	0.425
Urine protein >1^+^ *	162 (51.9%)	25 (39.0%)	48 (42.5%)	89 (65.9%)	<0.001
**Medication**					
Beta blockers (BB)	110 (35.3%)	18 (28.1%)	38 (33.6%)	54 (40%)	0.318
Calcium channel blockers (CCB)	190 (60.9%)	26 (40.6%)	60 (53.1%)	104 (77%)	0.02
RAAS blockers	177 (56.7%)	24 (37.5%)	63 (55.8%)	90 (66.6%)	0.04
**Diuretics Therapy**	144 (46.1%)	11 (17.2%)	35 (30.9%)	98 (72.6%)	
Mono Diuretic therapy	108 (34.6%)	11 (14%)	35 (32.7%)	62 (45.9%)	
• Loop diuretics	69 (22.1%)	7 (10.9%)	10 (8.8%)	52 (38.5%)	
• Thiazide Diuretics	39 (12.5%)	4 (6.3%)	25 (22.1%)	10 (7.4%)	
Multiple diuretic therapy	36 (11.5%)	0	0	36 (15.6%)	

Values of categorical variables are presented as percentages (%) whereas continuous variables are shown as mean ± SD or median with interquartile range.

P-value for continuous variables is calculated by one way ANOVA or Kruskal-Wallis H test, as appropriate, p-value for categorical variables is calculated by *X*^*2*^ test

P-value are calculated by comparing three groups (hypovolemic, hypervolemic and euvolemic)

*Assessment of urine protein was done via dipstick test

Mono diuretic therapy: either loop or thiazide diuretic, Multiple diuretic therapy: concomitant use of more than one diuretic

RAAS: renin-angiotensin aldosterone system blockers (include ACEI &ARBS)

**Fig 2:**
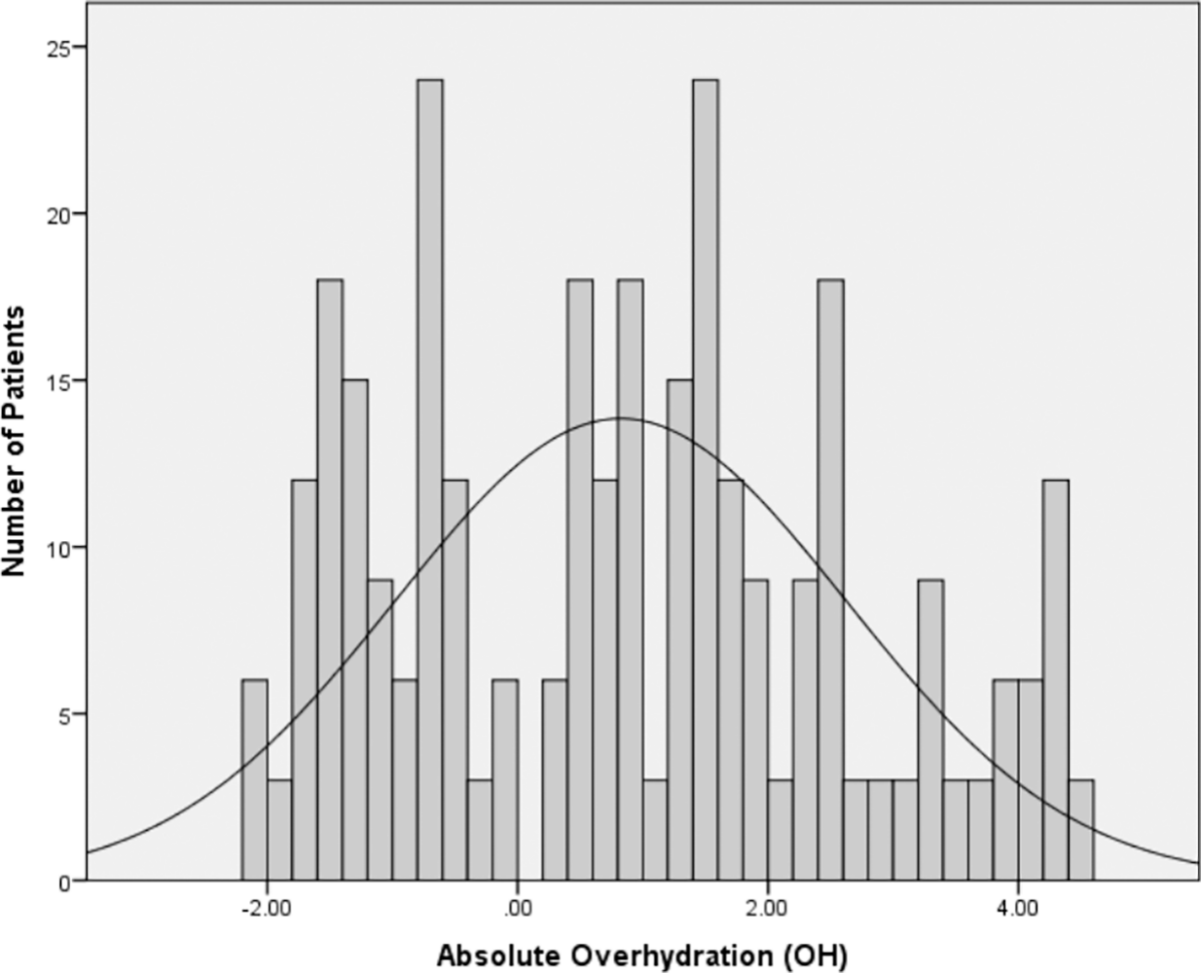
Distribution of absolute overhydration (0H) in 312 NDD-CKD patients, ranging between -2.1–4.4 L (82± 1.79).

Distribution of absolute overhydration (OH) in entire cohort is shown in Fig 2. On the basis of OH value, 64 (20.5%) patients were hypovolemic while euvolemia and hypervolemia were observed in 113 (36.2%) and 135 (43.3%) patients, respectively. Majority of the hypovolemic and euvolemic patients belonged to CKD stage 3 followed by CKD stage 4 while CKD stage 5 was more prevalent in hypervolemic group. Hyperlipidemia was found to be equally distributed among three categories of fluid status while all other comorbidities were more prevalent among hypervolemic patients. With the exception of β blockers, there was statistically significant difference in medication use of three groups, with hypervolemic patients receiving more medications than the two other groups. Inter-comparison between three groups showed that there was a step-wise increase in levels of extracellular water, total body water, systolic blood pressure, urinary protein excretion, glycated hemoglobin, serum uric acid, blood urea nitrogen (BUN) and leg edema score from hypovolemic to hypervolemic group. On the other hand, a stepwise decline was observed in lean tissue mass, serum hemoglobin, cholesterol and albumin from hypervolemic to hypovolemic group.

### Diuretics and Fluid Overload

A total of 153 (49.0%) patients received diuretics based on physician assessment of leg edema, blood pressure and cardiovascular complications. During follow-up period, diuretics were discontinued in 9 patients for clinical reasons; therefore these patients were excluded from final analysis leaving 144 (46.1%) patients receiving diuretics until end of follow-up. Baseline eGFR of patients receiving diuretics versus non-users is shown in Table 2. Out of 144 patients prescribed with diuretics, majority of the patients (72.6%) were hypervolemic while euvolemia and hypovolemia were observed in 35 (30.9%) and 11 (17.2%) patients, respectively. Loop diuretics were predominantly prescribed in hypervolemic patients [52 (38.5%)] while thiazide diuretics were received by euvolemic patients [25 (22.1%)]. Multiple diuretic therapies were observed in 36 (15.6%) patients and all of them were hypervolemic.

**Table 2:** Baseline value of eGFR in different fluid status categories with respect to diuretic use.

Fluid status category	eGFR ml/min/1.73m^2^ Diuretics users	eGFR ml/min/1.73m^2^ Diuretics non-users	P-value
Hypovolemic	28.9 ± 9.6	28.5 ± 10.1	0.26
Euvolemic	28.4 ± 10.8	29.8 ± 11.1	0.07
Hypervolemic	19.9 ± 8.2	21.5 ± 8.4	0.42

### Clinical Outcomes

In order to assess the impact of diuretics on clinical outcomes of patients, we categorized patients on the basis of diuretic use (Table 3) and assessed outcomes in terms of decline in eGFR, initiation of RRT and death. At the end of follow-up, the mean decline in eGFR of entire cohort was -2.5 ± 1.4 ml/min/1.73m^2^. Inter-comparison between diuretic users and non-users showed that decline in eGFR and initiation of RRT were more profound among diuretic users compared to non-users (Table 3).

**Table 3:** Comparison of outcomes between diuretic users and non-users.

Outcomes	Total cohort N = 312	Diuretic users N = 144	Non-users N = 168	p-value
**S-cr measurements**	4 (3–7)	5 (3–7)	5 (3–7)	
**eGFR**	23.7±7.1	22.3 ± 7.4	25.1 ± 6.8	<0.001
**ΔeGFR (ml/min/1.73m**^**2**^**)**	-2.5±1.4	-3.5 ± 1.6	-1.6 ± 0.77	0.02
**RRT**	36 (11.5%)	30 (20.8%)	6 (3.5%)	<0.001
**Death**	2 (0.6%)	2 (1.4%)	0	

p-value calculated by student t-test for continuous variables and chi-square test for categorical variables between diuretic users and non-users

S-cr: serum creatinine, ΔeGFR: change in estimated glomerular filtration rate. RRT: renal replacement therapy

Possible and well documented progression factors of GFR decline along with diuretic use were tested by Pearson`s correlation. Factors that showed significant correlation were subjected to multivariate regression analysis in order to find out independent determinants of GFR decline (Table 4). Systolic blood pressure, Diabetes mellitus, fluid overload, proteinuria, diuretics use and baseline eGFR were found to be significantly associated with GFR decline in current study.

**Table 4:** Determinants of eGFR decline in entire cohort (n = 312).

Variables	Univariate			Multivariate			
	Beta Coefficient (β)	R^2^	p-value	Beta Coefficient (β)	R^2^	Incremental R^2^	p-value
Age	-0.358	0.128	<0.001	-0.241	0.058	0.058	0.067
Male sex	-0.517	0.267	0.001	-0.283	0.080	0.136	0.082
Smoking	0.224	0.050	0.081	0.137	0.018	0.006	0.253
Systolic Blood pressure	-0.530	0.281	0.003	-0.368	0.135	0.104	0.012
Diabetes mellitus	-0.715	0.511	<0.001	-0.427	0.182	0.123	0.005
Cardiovascular disease	-0.217	0.047	<0.001	-0.092	0.008	0.042	0.142
Hyperlipidaemia	0.364	0.132	0.083	0.211	0.044	0.003	0.360
Fluid overload (OH)	-0.792	0.627	0.001	-0.497	0.247	0.122	0.003
Proteinuria	-0.699	0.489	<0.001	-0.562	0.315	0.041	<0.001
Diuretics	-0.583	0.340	<0.001	-0.384	0.147	0.062	0.016
Baseline eGFR	0.738	0.545	<0.001	0.544	0.295	0.071	<0.001

Multivariate Model R^2^ = 0.768

Continuous variables: age, systolic blood pressure, fluid overload, baseline eGFR

Dichotomous variables: male sex (0: female/1: male), smoking (0: non-smokers/1: smokers), diabetes mellitus (0: absent/1: present), cardiovascular disease (0: absent/1: present), Hyperlipidemia (0: absent/1: present), Proteinuria (0: absent/1: present), diuretics (0: non-users/1: users)

Multivariate regression analysis was performed by enter method

Renal replacement therapy (RRT) was initiated in 36 (11.5%) patients at the end of follow-up period. It is worthwhile to mention that among patients who initiated RRT, 30 patients were using diuretics. Majority of these patients (n = 28) initiated hemodialysis while peritoneal dialysis was chosen by 8 patients. With respect to CKD staging, out of 36 patients who progressed to RRT, 19 (63.3%) belonged to CKD stage 5 while 11 patients (36.7%) had CKD stage 4. Fortunately, none of the patient in CKD stage 3 initiated RRT. Mortality was observed in two cases and it is interesting to note that these patients belonged to CKD stage 5 hypervolemic group receiving diuretic therapy. Cardiogenic shock was the documented cause of fatality in both cases. We did not observe any death among patients with CKD stage 3 and stage 4.

We further performed multivariate regression analysis to evaluate risk of initiation of dialysis and eGFR decline with respect to diuretic use. The unadjusted and adjusted risk of commencing RRT in diuretic users and non-users is shown in Table 5. Regardless of fluid status category, diuretic users had increased risk of RRT initiation and eGFR decline than non-users.

**Table 5:** Hazard ratio of diuretic users and non-users in relation to renal outcomes.

Renal outcomes	Unadjusted univariate HR (95% CI)	p-value	Adjusted multivariate HR (95% CI)	p-value
**Initiation of RRT**				
Non-users (Reference)	1		1	
Diuretic users	3.4 (1.29–2.56)	<0.001	2.5 (1.69–3.12)	0.03
**Decline in eGFR**				
Non-users (Reference)	1		1	
Diuretic users	5.6 (2.57–4.83)	<0.001	3.8 (2.32–4.61)	0.01

Model adjusted for fluid status, systolic BP, baseline eGFR as continuous variable

CV disease, proteinuria, diabetes mellitus were adjusted as categorically

## Discussion

Current study evaluated fluid status in a cohort of 312 NDD-CKD patients by using noninvasive technique of bioimpedance spectroscopy (BIS). By using cutoff values of absolute overhydration (OH), study subjects were divided into 3 categories of fluid status i.e. hypovolemia, euvolemia and hypervolemia. A total of 135 (43.2%) patients met the definition criteria of hypervolemia that is less than to what reported in different studies. Reason for low prevalence of hypervolemia in our study might be attributed to difference in cutoff values of fluid overload. On the basis of absolute OH range (0.9–4.1L), maximum prevalence of hypervolemia (65%) was reported by Tsai et al [12]. We did not include 0.9 to 1.1 L in our cutoff range. Similarly in other studies hypervolemia was reported to be 52% and 54.6% based on OH > 7% and OH/ECW ≤ 0.15 respectively [11, 13]. Although the prevalence of hypervolemia reported in our study is less than to what reported in other studies, still 43% of our study subjects were hypervolemic indicating approximately half of NDD-CKD patients are not getting adequate care, despite of their regular visits to nephrology clinic where much attention is paid to volume status. These findings suggest that current clinical and technical tools for aiding physicians to diagnose fluid status and achieve euvolemia are not sufficient. An additional non-invasive tool such as BCM can be useful for routine clinical use in hospitals and would effectively aid diagnosis of fluid overload in NDD-CKD patients.

A total of 144 (46.1%) patients received diuretics at baseline. Apart from management of hypertension and peripheral edema, diuretics are prescribed to control fluid overload [3]. It is interesting to note that diuretics were found to be associated with adverse renal outcomes in our study cohort. Poor renal outcomes among diuretic users might be contributed to several factors. Firstly, maximum diuretic use was observed in hypervolemic patients. As evident from the term “hypervolemia”, these patients were having higher ECW as compared to the other two groups. Hypervolemia itself causes eGFR decline by independently effecting vascular and endothelial cells leading to artheosclerosis and arterial stiffness [21]. Moreover, fluid overloaded patients have increased extravascular volume and decreased intravascular volume leading to decrease blood flow towards kidney [22, 23]. In addition, a recent study has shown strong association between fluid overload and proteinuria. As proteinuria significantly causes decline in eGFR, this might add to another possibility of poor renal outcomes in hypervolemic patients [24]. Secondly, systolic blood pressure (SBP) was significantly higher among these patients causing more use of other classes of anti-hypertensives. Both RAAS and CCB alter intra-glomerular hemodynamics leading to nephrotoxicity [25, 26]. However, the attribution of hypervolemia, proteinuria, higher SBP and use of anti-hypertensives was adjusted in all statistical analysis (Tables 3–5). Despite adjustment of these confounders, diuretics were found to be independently associated with poor renal outcomes indicated by decline in eGFR and initiation of RRT in NDD-CKD patients (Tables 4 and 5).

Previously, a number of evidences have reported that diuretic therapy is either detrimental to renal function or it significantly allows quantifiable renal impairment [4, 9, 27]. Secondary analysis of major hypertensive trials have shown that diuretics cause significant increase in serum creatinine values [28–30]. Data analysis of NHANES III survey reported that increase creatinine level is directly proportional to diuretic prescriptions [31]. Similar results were reported by Hawkins and Houston in their retrospective analysis of United States Renal Data System (USRDS). By using data fusion methodology, authors found that increase diuretic distribution is directly associated with ESRD incidence [32]. Similar few small sample size observational studies conducted in CKD patients have also reported rise in serum creatinine with diuretic use [9].

To the best of our knowledge, our study is first hospital based clinical study demonstrating association of diuretic use with decline in eGFR and initiation of RRT in NDD-CKD patients. The exact mechanism by which diuretics cause renal injury is not clear. Apart from metabolic disturbances, diuretics directly cause apoptosis in distal tubular cells of nephron [33]. Hypokalemia that is the major metabolic disturbance of both thiazide and loop diuretics leads to renal hypertrophy and tubulointerstitial fibrosis. Concomitant administration of more than one diuretic causes massive volume loss that results in renal vasoconstriction with increased tubular uptake of sodium chloride (NaCl) and decreased urine output (UO). Prolonged vasoconstriction then leads to tubular dysfunction and necrosis [34]. Moreover, co-administration of diuretics with other anti-hypertensives especially vasodilators (calcium channel blockers) results in rapid fluctuations of BP causing pre-renal azotemia [33].

The purpose of current study is not to the challenge the potential benefits of diuretics in NDD-CKD patients. There is a well reported evidence for potential benefits of diuretics as not only anti-hypertensive agents but they also significantly reduce risk of cardiovascular and cerebrovascular diseases. However, we made an attempt to highlight the potential risks of diuretics with an intent to underscore clinicians’ attention towards dark side of diuretic therapy that can be potentially fatal for patients in long run. Need of the hour is careful selection of hypervolemic NDD-CKD patients where benefit of using diuretic therapy outweighs subsequent risks and use of other equally rational treatments (CCB, RAAS blockers).

### Limitation

Some potential limitations of current study are needed to be addressed. Being an observational study, confounding by indication was the main limitation. Efforts were made to minimize bias by adjusting confounders in all statistical analysis. Fluid status was measured once only at the time of beginning of study, therefore changes in fluid status over time were not considered. Secondly, BCM device has not been validated in CKD patients but previous studies in CKD population has shown that there is a linear relationship between leg edema score and severity of fluid overload as assessed by BCM device. Therefore, it is widely assumed in clinical practice that BCM gives appropriate results of fluid status in CKD patients. Furthermore, fluid overload might lead to underestimation of creatinine levels and therefore gives misleading creatinine values in hypervolemic patients. Use of diuretics was recorded categorically, not as time varying dose of medication. Our study lacks information regarding salt consumption and dietary intake, as both of them affect fluid status and diuretic efficacy; therefore it might affect study results. Lastly, the follow-up period was only 12 months. A longer follow-up period will give better understanding of outcomes of diuretics especially in terms of decline in eGFR and disease progression. Despite mentioned limitations, current study is strengthened by its prospective nature and being first study in Asia that shows association between diuretic use and decline in renal function in clinical settings.

## Conclusions

In conclusion, current study demonstrates that diuretic use is an independent predictor of adverse renal outcomes in NDD-CKD patients causing decline in eGFR and increasing the risk of RRT initiation. Unless a contradictory data from randomized controlled trial discourages above findings, it is cautiously concluded that irrespective of fluid overload, diuretics cause adverse renal outcomes. Future interventional studies or double blinded randomized controlled studies with large sample size are needed to rule out the association between diuretic use (type and dose) and renal outcomes in NDD-CKD patients, as this complicated triangle is present in majority of NDD-CKD patients. Such studies should be designed to include time averaged defined daily dose of diuretics.
